# Nonspreading Rift Valley Fever Virus Infection of Human Dendritic Cells Results in Downregulation of CD83 and Full Maturation of Bystander Cells

**DOI:** 10.1371/journal.pone.0142670

**Published:** 2015-11-17

**Authors:** Nadia Oreshkova, Paul J. Wichgers Schreur, Lotte Spel, Rianka P. M. Vloet, Rob J. M. Moormann, Marianne Boes, Jeroen Kortekaas

**Affiliations:** 1 Department of Virology, Central Veterinary Institute, part of Wageningen University and Research Centre, Lelystad, The Netherlands; 2 Department of Infectious Diseases and Immunology, Virology Division, Faculty of Veterinary Medicine, Utrecht University, Utrecht, The Netherlands; 3 Department of Pediatric Immunology and Laboratory of Translational Immunology, University Medical Centre Utrecht/Wilhelmina Children’s Hospital, Utrecht, The Netherlands; George Mason University, UNITED STATES

## Abstract

Vaccines based on nonspreading Rift Valley fever virus (NSR) induce strong humoral and robust cellular immune responses with pronounced Th1 polarisation. The present work was aimed to gain insight into the molecular basis of NSR-mediated immunity. Recent studies have demonstrated that wild-type Rift Valley fever virus efficiently targets and replicates in dendritic cells (DCs). We found that NSR infection of cultured human DCs results in maturation of DCs, characterized by surface upregulation of CD40, CD80, CD86, MHC-I and MHC-II and secretion of the proinflammatory cytokines IFN-β, IL-6 and TNF. Interestingly, expression of the most prominent marker of DC maturation, CD83, was consistently downregulated at 24 hours post infection. Remarkably, NSR infection also completely abrogated CD83 upregulation by LPS. Downregulation of CD83 was not associated with reduced mRNA levels or impaired CD83 mRNA transport from the nucleus and could not be prevented by inhibition of the proteasome or endocytic degradation pathways, suggesting that suppression occurs at the translational level. In contrast to infected cells, bystander DCs displayed full maturation as evidenced by upregulation of CD83. Our results indicate that bystander DCs play an important role in NSR-mediated immunity.

## Introduction

Rift Valley fever virus (RVFV) replicon particles, also known as nonspreading RVFV (NSR), resemble authentic RVFV by structure and infectivity [1]. They retain the genes encoding proteins necessary for viral RNA amplification, but are deprived of the gene encoding the structural glycoproteins, required for the generation of progeny virions. In addition, NSR particles lack the gene encoding the nonstructural NSs protein, which counteracts innate immune responses [2–5]. The absence of the NSs gene adds to the safety profile of NSR and provides an expression slot for a protein of interest. These combined features render NSR an intrinsically safe and powerful platform for the development of vaccines.

NSR proved to be highly efficacious when used as a RVF vaccine both in mice and in sheep, the latter being the main natural target species of the virus [1, 6]. A single vaccination with similar replicon particles, developed by Dodd and co-workers, resulted in systemic induction of interferon-stimulated genes as early as 12 h post vaccination and initiation of an antiviral state that protected mice from lethal RVFV challenge already 24 hours post vaccination [7]. The efficacy of the NSR vaccine was further improved by introducing in the NSR genome the gene encoding the glycoprotein Gn, which is the dominant target of neutralizing antibodies. A single vaccination with the resulting NSR-Gn vaccine provided sterile protection against RVFV challenge in lambs [8, 9]. More recently, we developed NSR particles encoding the hemagglutinin (HA) of the influenza virus. These particles protected mice from a lethal dose of influenza virus after a single intranasal or intramuscular administration [10]. Vaccination with NSR was consistently associated with neutralizing antibody responses and robust T-cell responses with strong Th1 polarization [1, 6, 8–10]. The ability of NSR to induce strong cellular immune responses was recently confirmed by controlling outgrowth of tumor cells in mice by vaccination with NSR particles that expressed a single tumor-associated CD8-restricted epitope [11]. The remarkable efficacy of the NSR vaccine prompted further studies on the molecular basis of NSR-mediated immunity.

Recent findings by Lozach *et al* demonstrated that wild-type RVFV can efficiently infect human DCs, using dendritic cell-specific intercellular adhesion molecule-3-grabbing non-integrin (DC-SIGN) as a receptor [12]. Infection of DCs resulted in generation of high titers of progeny virions. In another study, RVFV was shown to specifically target cells of the monocyte/macrophage/dendritic cell lineages in mice [13]. These data suggest that the interaction of RVFV with DCs plays an important role in the pathogenesis of RVF. Innate immune responses resulting from RVFV infection of bone marrow-derived macrophages are efficiently counteracted by the NSs protein [14], and it is plausible that NSs has a similar function in DCs. However, infection of DCs with NSR particles lacking NSs should result in full-blown antiviral responses, which likely contribute to vaccine efficacy.

DCs are key players in the initiation and regulation of immune responses. Immature DCs are equipped with a broad range of pattern recognition receptors and are very effective in recognizing various pathogen-associated molecular patterns (PAMPs). When contact with a PAMP occurs, DCs start to mature. During this process, the cells undergo changes in their morphology, migratory capability, expression of surface molecules and function [15]. The cells migrate from areas of antigen uptake to T-cell areas of secondary lymphoid organs, where they present antigen-derived peptides and instruct epitope-specific naïve T-cells to develop their effector function [16]. The maturation of DCs is associated with increased expression of surface molecules, such as MHC-I and MHC-II, which are involved in antigen presentation, as well as CD86, CD80, CD40 and CD54, which act as co-stimulators in T-cell activation [17, 18]. The most characteristic marker of fully matured human DCs is CD83 [19, 20]. Although the exact mechanism of action and the specific ligand of CD83 remain to be elucidated, surface expression of this molecule on DCs is critical for priming naïve T cells [21, 22].

In the present study, we investigated the interaction between NSR and human DCs. We found that DCs are efficiently infected and tolerate viral genome replication and protein expression. The cells exhibited evidence of maturation, manifested by morphological changes, secretion of the proinflammatory cytokines IFN-β, IL-6 and TNF and upregulation of the surface molecules CD40, CD80, CD86, MHC-I and MHC-II. Surprisingly, while bystander DCs displayed upregulation of CD83, suggestive of full maturation, infected DCs exhibited a gradual downregulation of CD83. This effect was not associated with corresponding downregulation of CD83 mRNA or defects in mRNA transport from the nucleus. Neither the proteasomal nor the endocytic degradation pathways seemed to be involved in the decrease in CD83 levels, suggesting that NSR-mediated downregulation of CD83 expression occurs at the translational level. The incomplete maturation of NSR-infected DCs and full maturation of bystander DCs suggest that only the latter play an active role in NSR-mediated immunity.

## Materials and Methods

### Ethics Statement

Blood samples were collected from healthy donors at the Laboratory of Clinical Chemistry and Haematology of the University Medical Center (UMC, Utrecht, The Netherlands). Donors provided written consent for use of the material for scientific research. The use of the material was approved by the Medical Ethics Committee of the UMC under protocol number 07-125/C.

### Cells

Peripheral blood mononuclear cells (PBMCs) were isolated from the blood of healthy donors by ficoll isopaque density gradient centrifugation (GE Healthcare Bio-Sciences AB) and frozen until use. PBMCs were used as a source of monocyte-derived dendritic cells (MoDCs). Immature DCs were cultured as previously described [23]. Briefly, PBMCs were seeded in standard 48- or 96-well culture plates in X-VIVO-15 medium with gentamycin (Lonza), supplemented with 2% heat-inactivated and 0.2 μm-filtered FCS (Bodinco). Cells were allowed to adhere for 1 h. Non-adherent cells were subsequently removed by washing with PBS and adherent cells were cultured in serum-free X-VIVO-15, supplemented with 450 U/mL GM-CSF and 300 U/mL IL-4 (Milteny). Medium was refreshed on the second day of incubation. On day 5, cells were stimulated with LPS, NSR or NSRmock diluted in RPMI 1640 with HEPES and glutamine (Gibco), supplemented with 10% FCS. Medium was used for negative controls. The stimulation conditions were selected for optimal infectivity of NSR. DCs were harvested at different time points after stimulation as indicated in the results section, by replacing the growth medium with cold PBS, followed by shaking (450 rpm) of the culture plates for 1 h at 4°C to detach cells.

### Generation of nonspreading RVFV (NSR) and control inoculum (NSRmock)

NSR particles were generated as previously described [8]. Briefly, NSR replicon cell lines were transfected with a plasmid encoding the RVFV surface glycoproteins Gn and Gc. NSR containing supernatants were harvested the next day and cleared from cell debris by centrifugation at 4,500 x *g* for 15 min. Subsequently NSR particles were purified and concentrated by ultracentrifugation at 64,000 x *g* for 2.5 h on a 2 ml 25% sucrose cushion, followed by resuspension in Opti-MEM (Invitrogen), supplemented with 0.2% heat-inactivated FCS. Particles were stored at -80°C until use. For generation of NSRmock, a similar procedure was used, but the replicon cells were transfected with a plasmid that encodes only the Gc protein. Supernatants harvested after this transfection contain the same media, transfection reagents and cellular metabolism products, but lack infectious NSR particles. The absence of infectious particles in NSRmock control preparation was confirmed by titration on BHK21 cells.

### Flow cytometry

The cell-surface phenotypes of unstimulated and stimulated DCs were analysed by flow cytometry using human-specific mAbs: anti-CD40 (clone 5C3) and anti-CD83 (clone HB15e) (eBioscience); anti-CD80 (clone L307.4), anti-CD86 (clone IT2.2) and anti-CD11c (clone B-ly6) (BD); anti-HLA-A,B,C (clone W6/32) and anti-HLA-DR (clone L243) (BioLegend), together with the respective isotype controls. Optimal concentrations of the antibodies were determined prior to flow cytometry assays. The DC population was selected by gating on cells that were double positive for CD11c and MHC-II. Median fluorescence intensity (MFI) was used as measure for expression of the analysed molecules. Data was acquired using Canto II flow cytometer (BD) and analysed using FlowJo software.

### Cytokine assay

PBMCs were seeded in 48-well culture plates and after an initial adherence step for 1 h as described above, were washed vigorously with PBS so that only adhering cells were retained. After 5 days of differentiation, DCs were stimulated with LPS, NSR, NSRmock or medium and after 24h supernatants were harvested, pre-cleared by slow-speed centrifugation and stored at -80°C until use. Concentrations of IFN-β, TNF, IL-6 and IL-10 were determined using a multiplex assay (eBioscience), according to the manufacturers’ instructions using the Luminex 200 system.

### Polyacrylamide gel electrophoresis (PAGE) and Western blotting

DCs were harvested 24 h post stimulation, counted and brought to equal concentrations in Pierce IP lysis buffer (Thermo Scientific), supplemented with protease inhibitors (Roche). Samples containing 50,000 cells were either directly denatured in standard Laemmli sample buffer or first pre-treated with peptide-N-Glycosidase F (PNGase F, BioLabs^®^ Inc.) according to the manufacturers’ instructions. Proteins present in cell lysates were separated by sodium dodecyl sulfate polyacrylamide gel electrophoresis (SDS-PAGE), followed by Western blotting as previously described [10]. Rat monoclonal anti-human CD83 antibody (clone 1G11, Enzo Life Sciences) and corresponding secondary horseradish peroxidase (HRP)-conjugated antibody were used to detect CD83. The blot with samples without PNGase F treatment was subsequently stripped and re-stained with mouse monoclonal anti-human glyceraldehyde-3-phosphate dehydrogenase (GAPDH) (clone 0411) and anti-GFP (clone B-2) antibodies (Santa Cruz) and corresponding secondary HRP-conjugated antibodies.

### ELISA to detect soluble CD83

Supernatants from DCs cultures, stimulated with LPS or NSR were harvested and centrifuged at 3,000 x *g* for 10 min to remove cell debris. Concentrations of soluble CD83 were determined with a commercial ELISA kit (Sino Biological Inc.) according to the manufacturers’ instructions. Each sample was tested at two different concentrations in triplicate. A standard curve was generated using serial dilutions of recombinant CD83, provided with the kit.

### Proteasome inhibition assay

DCs were stimulated with different stimuli for 8 h after which clasto Lactacystin β-lactone (CLBL) was added at a final concentration of 5 or 10 μM. The solvent of CLBL, dimethylsulfoxide (DMSO), was used as a control. Sixteen h after these treatments, cells were harvested and analysed by flow cytometry. Alternatively, cells were lysed and used for detection of total amounts of CD83 by SDS-PAGE and Western blotting. Control staining for GAPDH and GFP was performed after stripping the blot. Procedures and antibodies are described above.

### Endocytosis inhibition assay

DCs were stimulated with LPS+NSRmock, LPS+NSR or medium, for 6 h or 12 h. Cytochalasin D (Sigma) was subsequently added to a final concentration of 10 μg/ml. The solvent of Cytochalasin D, dimethylsulfoxide (DMSO), was used as a control. Cells were harvested 6, 12 or 18 h after Cytochalasin D/DMSO treatment and analysed by flow cytometry.

### RNA isolation and real-time PCR

After stimulation of DCs for 24 h, culture medium was discarded and cells were immediately lysed with Trizol. Total RNA was isolated with the Direct-zol™ RNA MiniPrep kit (Zymo research) according to the instructions of the manufacturer. 100 ng RNA of each sample was subsequently reverse-transcribed using random primers and Superscript III reverse transcriptase (Promega). Quantitative real-time PCR was performed as previously described [24]. The primer sequences are provided in Table in S1 Table.

### Single-molecule RNA fluorescence *in situ* hybridization (FISH)

DCs were cultured on a CultureWell™ 16 Chambered Coverglass (C-37000, Grace Bio-labs) and were stimulated with LPS, NSR or were left unstimulated. In a control FISH assay, cells were stimulated with LPS or simultaneously treated with LPS and 0.1 μg/ml Actinomycin D or 10 nM Leptomycin B (Enzo Life Sciences). 24 h post stimulation, cells were fixed with 4% paraformaldehyde (10 min) and permeabilized with 70% ethanol (>1 h at 4°C). Individual intracellular GAPDH, CD80 and CD83 mRNAs were subsequently visualized using human specific CD80, CD83 and GAPDH cDNA probes labelled with quasar 570 (Table in S2 Table) according to the Stellaris® FISH method (protocol for adherent cells, Biosearch Technologies) [25]. The oligonucleotides for GAPDH were predesigned by Biosearch Technologies, whereas the oligonucleotides for CD80 and CD83 were designed using the online Stellaris probe design software. Images were generated using an Axioskop 40 (Zeiss) fluorescent microscope with a 1.28 NA 100× oil objective and an AxioCam MRm camera. Raw images were deconvolved and analysed using Huygens software (Scientific Volume Imaging, Hilversum, The Netherlands). Individual spots were counted with dedicated online software (StarSearch, http://rajlab.seas.upenn.edu/StarSearch/launch.html).

### Statistical analyses

Differences in expression levels of surface markers CD40, CD80, CD83, CD86, MHC-I and MHC-II were evaluated with repeated measures one-way analysis of variance (ANOVA) with Dunnett’s post hoc test. Differences in spot numbers corresponding to CD80, GAPDH and CD83 mRNAs in the FISH assay were analysed with one-way ANOVA with Dunnett’s post-hoc test. Differences in quantities of soluble CD83 were analysed with Welch ANOVA with Games-Howell post hoc test. Differences in secreted cytokines, as well as PCR-determined mRNA levels of CD80, CD83, GAPDH and peptidylprolyl isomerase A (PPIA) were analysed using a Student’s T test. Student’s T test with Bonferonni correction for multiple comparisons was used also to evaluate differences in CD83 expression in the endocytosis inhibition assay using the CytD inhibitor. Values of p<0.05 were considered significant. Analyses were performed with GraphPad Prism® 5 or IBM SPSS stastistics 20 software.

## Results

### NSR infection of human DCs triggers phenotypic maturation

**Fig 1:**
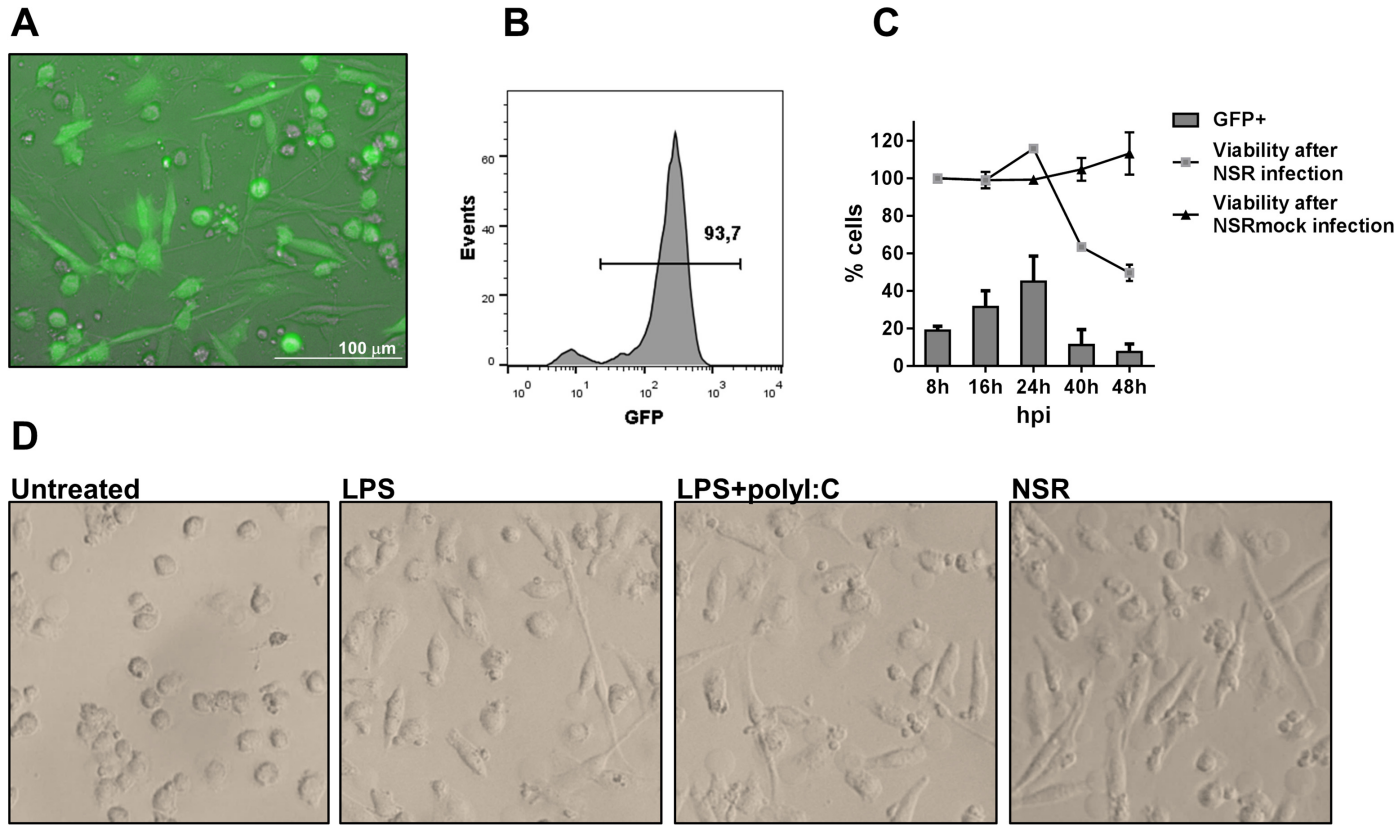
Infection of DCs by NSR. (A) DCs were infected with NSR for 24 h and evaluated for expression of GFP, using an EVOS fluorescence microscope. (B) Infection efficiency under optimal conditions as determined by flow cytometry. (C) Viability and percentage of infected cells at different time points after infection. Cells were infected with NSR or mock-infected with NSRmock, harvested at the indicated time points, stained with 7AAD and analysed by flow cytometry. The percentage of GFP expressing cells (bars) and the viability after NSR or NSRmock infections (lines) is depicted. Viability of the cells was calculated relative to the viability at 8 hpi, which was set at 100%. The data depict average values from two experiments with cells from two different donors ±SD. (D) Morphology of DCs stimulated with the indicated stimuli at 24 h post treatment.

Immature DCs were generated as described in materials and methods and were infected with NSR particles expressing GFP (Fig 1A). Initial experiments revealed that GFP expression in DC cultures was detectable already at 6 h post infection (hpi, data not shown). Infection efficiencies varied between different experiments and donors, but exceeded 90% under optimal conditions (Fig 1B).

Monitoring of NSR infection in time revealed that the number of GFP-positive (GFP+) DCs increased gradually until 24 hpi (Fig 1C). After that time point, a rapid decrease was noticed and at 48 hpi a 5-fold reduction was observed, compared to GFP+ cell counts at 24 hpi. The decrease in GFP+ cells coincided with a decrease in the total numbers of viable cells, while viability of NSRmock-infected control cells remained stable over time.

Infection of DCs with NSR resulted in distinct morphological changes in the cells. Infected DCs acquired flat and stretched shapes, discriminating them from unstimulated cells, which remained predominantly round-shaped (Fig 1D). The phenotype of the infected cells resembled closely that of cells stimulated with LPS or a combination of LPS and poly(I:C), which are known to trigger DCs maturation [26]. This finding suggests that NSR infection of DCs results in maturation.

### Infected DCs secrete proinflammatory cytokines

**Fig 2:**
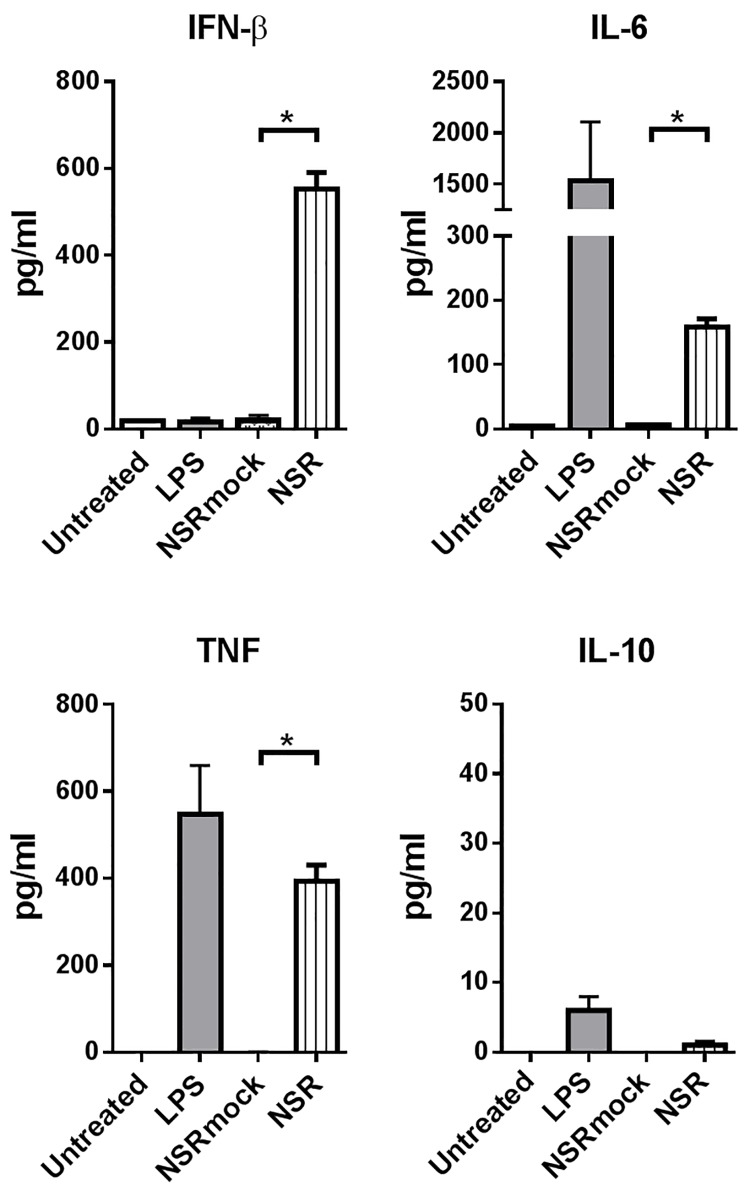
Cytokine secretion by NSR-infected DCs. Supernatants of infected or control-treated DCs were harvested at 24 hpi and analysed with a luminex-based cytokine assay. Bars represent the mean cytokine concentrations ± SD of triplicates with cells from one donor. Statistical significance between infected (NSR) and mock-infected (NSRmock) conditions is indicated.

To evaluate whether NSR infection results in the induction of a proinflammatory cytokine response, supernatants of infected DCs were analysed for the presence of IFN-β, TNF, IL-6 and IL-10. Infection was performed such that more than 90% of the cells were positive for GFP. As expected, control LPS stimulation resulted in the induction of IFN-β, TNF and IL-6, while NSRmock-infected cells did not show any induction of cytokines (Fig 2). NSR-infected DCs showed relatively strong induction of IFN-β, TNFα and IL-6. In contrast, concentrations of IL-10, which is known for its immunosuppressive functions [27, 28] remained low. These results show that NSR is capable of inducing proinflammatory cytokine responses in DCs.

### NSR-infected DCs upregulate maturation markers, but only bystander DCs upregulate CD83

Maturation of DCs is associated with upregulation of the surface expression of MHC class I and II molecules and co-stimulatory molecules such as CD80, CD86 and CD40 [17, 18]. The hallmark of fully matured human DCs is cell surface presentation of CD83. To test the maturation status of NSR infected DCs, we analysed surface expression of MHC-I, MHC-II, CD40, CD80, CD83 and CD86 molecules upon infection.

**Fig 3:**
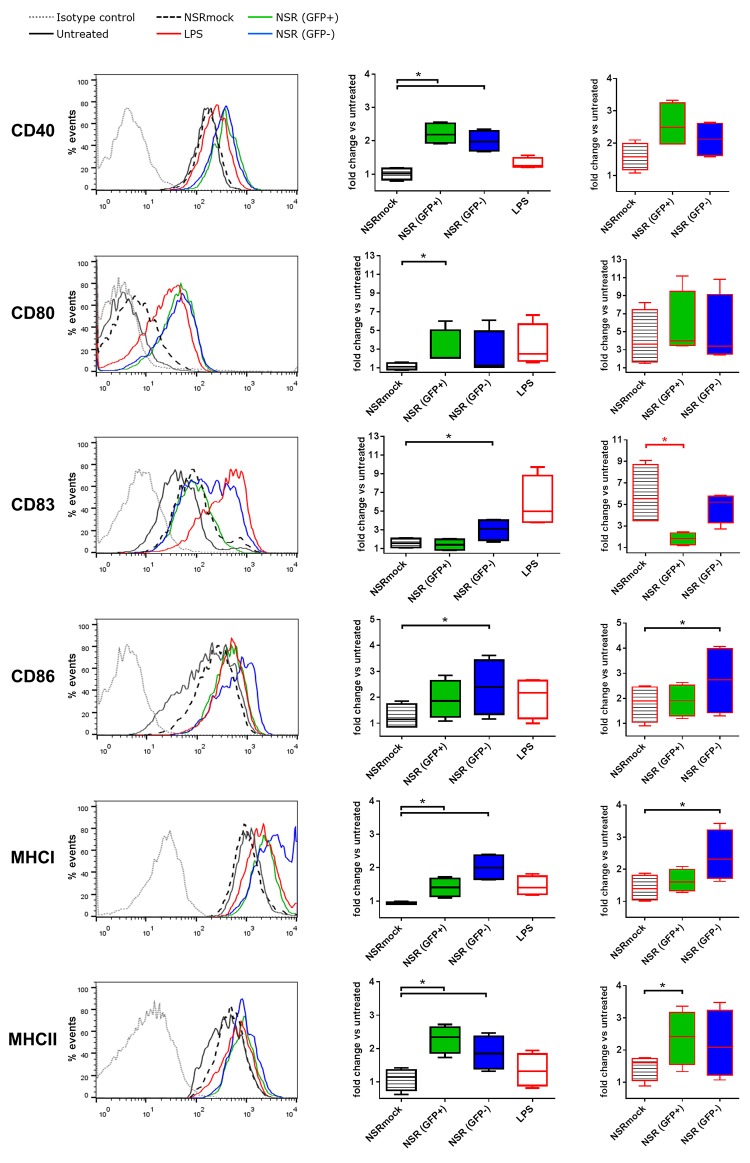
Surface expression of CD40, CD80, CD83, CD86, MHC-I and MHC-II on DCs at 24 h after NSR infection as measured by flow cytometry. Immature DCs were infected with NSR, mock-infected with NSRmock, or stimulated with LPS (left and middle panels). Alternatively, cells were infected with NSR or mock-infected with NSRmock in the presence of LPS (right panels). The left panel shows representative histograms of surface marker measurements on cells stimulated with LPS, mock infected cells (NSRmock), cells infected with NSR (GFP+) and uninfected bystander DCs (GFP-). Expression of markers in untreated cells and an irrelevant isotype control are depicted. The middle and right panels represent average data from 4 independent experiments performed with cells from 3 donors. The box plots depict MFI of the different markers relative to untreated cells. A black asterisk indicates upregulation compared to the control and a red asterisk indicates downregulation.

Flow cytometry analysis of infected GFP+ DCs at 24 hpi revealed a significant induction of the surface expression of CD40, CD80, MHC-I and MHC-II as compared to cells incubated with NSRmock (Fig 3, left and middle panels). The increase in surface expression of CD86 was not significant. Strikingly, although expression of CD83 was upregulated 4- to 10-fold at 24 h after LPS stimulation, no upregulation of CD83 was observed in GFP+ cells at this time point.

To evaluate whether NSR infection can counteract LPS-induced CD83 expression, a co-stimulation with LPS and NSR was performed. Comparable surface expression levels of CD40, CD80, CD86 and MHC-I were found in DCs co-stimulated with NSR+LPS or control-stimulated with NSRmock+LPS. However, co-stimulation of DCs with LPS and NSR did not result in upregulation of CD83 (Fig 3, right panels). From these results it can be concluded that NSR actively downregulates CD83 surface expression. Upon addition of LPS, GFP- cells displayed comparable amounts of surface expressed CD40, CD80, CD83 and MHC-II markers and increased amounts of CD86 and MHC-I.

Analysis of DCs that were GFP-negative (GFP-) at 24 hpi, revealed that the levels of all surface molecules except CD80 were significantly elevated as compared to NSRmock stimulated cells, presumably representing a bystander effect resulting from cytokines released by GFP+ cells (Fig 3). Addition of LPS resulted in further upregulation of CD86 and MHC-I.

### Kinetics of CD83 expression in NSR-infected DCs

**Fig 4:**
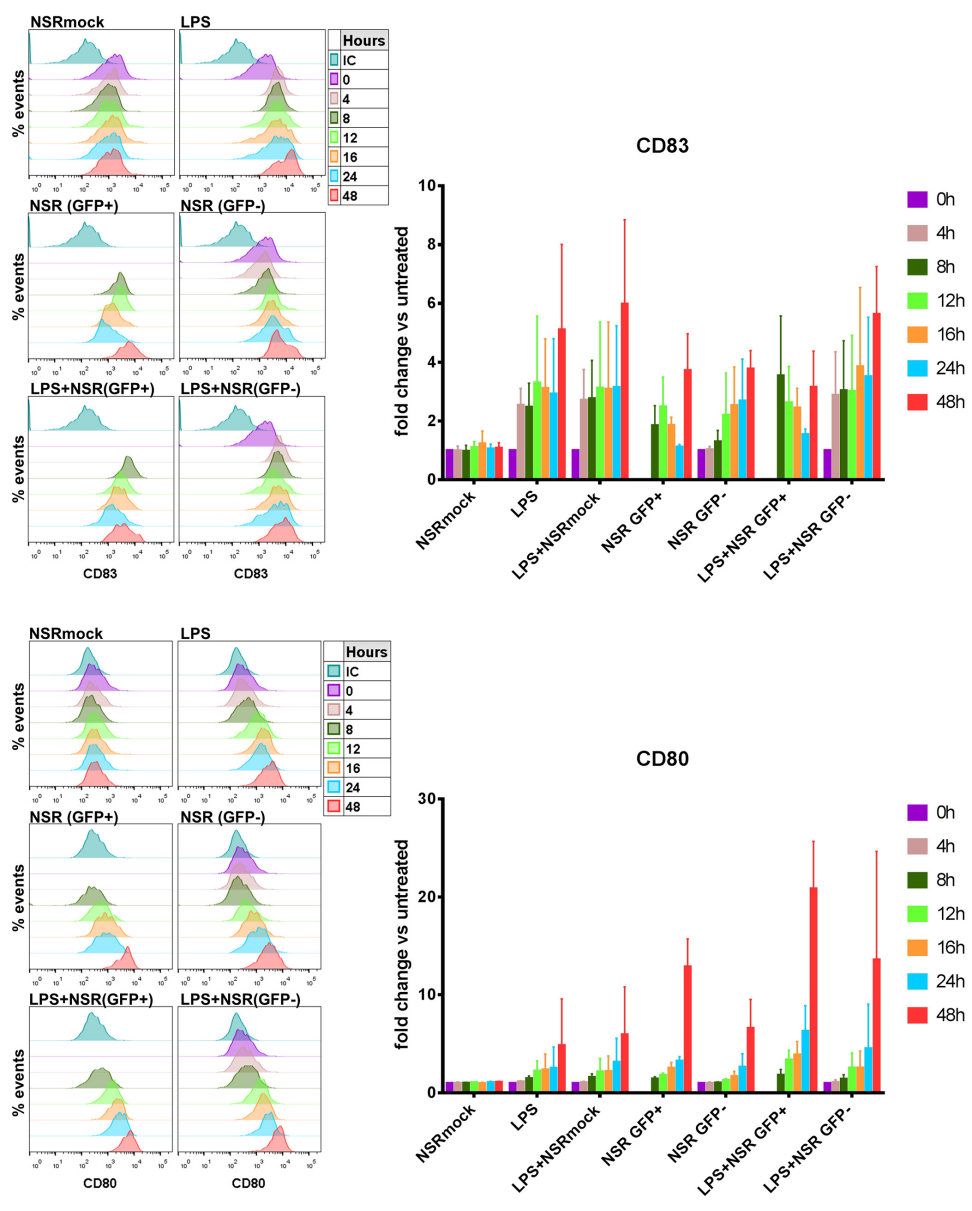
Analysis of CD83 and CD80 surface expression in time. DCs were treated with NSRmock, NSR, LPS, LPS+NSRmock or LPS+NSR and were harvested at 0, 4, 8,12, 16, 24 and 48 h post treatment. Surface expression of CD83 (upper panels) and CD80 (lower panels) were measured by flow cytometry. Left panels show histograms from one representative experiment. Time points are depicted with different colors and the color code is shown at the right. IC–isotype control. Right panels illustrate average data from three independent experiments with cells from three different donors. Bars represent means ±SD of the fold change of MFI relative to untreated cells.

To investigate the dynamics of CD83 surface expression in more detail, DCs were harvested after 4, 8, 12, 16, 24 and 48 hpi and analysed with flow cytometry. As a reference marker for upregulation, CD80 was used. NSRmock stimulated cells did not display changes in CD83 surface expression during the whole observation period, while LPS-stimulated cells displayed upregulation of CD83 already after 4 h and expression levels remained high until the end of the observation period (Fig 4, upper panel). Similar expression levels were observed after combined LPS+NSRmock treatment. In NSR-infected cells, the GFP signal was detectable at 8 hpi. In GFP+ cells, an initial upregulation of CD83 was observed which peaked at 12 hpi. After that time point, CD83 levels decreased gradually and at 24 hpi reached the levels of the NSRmock control. Contrastingly, in GFP- cells, CD83 expression levels were initially comparable to those of control cells and at 12 hpi levels increased and remained high, revealing that CD83 upregulation in bystander DCs depended on the presence of GFP+ cells. When a combination of LPS and NSR was used to stimulate DCs, maximal upregulation of CD83 in GFP+ cells was reached already at 8 hpi, consistent with the presence of LPS. However, as time progressed, CD83 expression decreased, reaching the lowest levels at 24 hpi. In GFP- cells, CD83 levels resembled those in cells stimulated with LPS and LPS combined with NSRmock. These data indicate that infection with NSR results in initial upregulation of CD83, which is augmented by the presence of LPS. Later, as viral genome replication progresses, CD83 is gradually depleted from the cell surface, counteracting the effect of LPS. Notably, CD83 was dramatically upregulated at 48 hpi in all stimulated cells, including those positive for GFP.

In contrast to CD83, CD80 displayed a gradual upregulation in cells infected with NSR and in cells stimulated with a combination of LPS and NSR, regardless of the expression of GFP. The dynamics of CD80 expression in GFP+ cells resembled closely that in cells stimulated with LPS or LPS+NSRmock (Fig 4, lower panel). A strong increase in the surface expression of CD80 was observed at 48 hpi similar to CD83.

### CD83 mRNA levels and subcellular distribution are unaffected by NSR-infection

**Fig 5:**
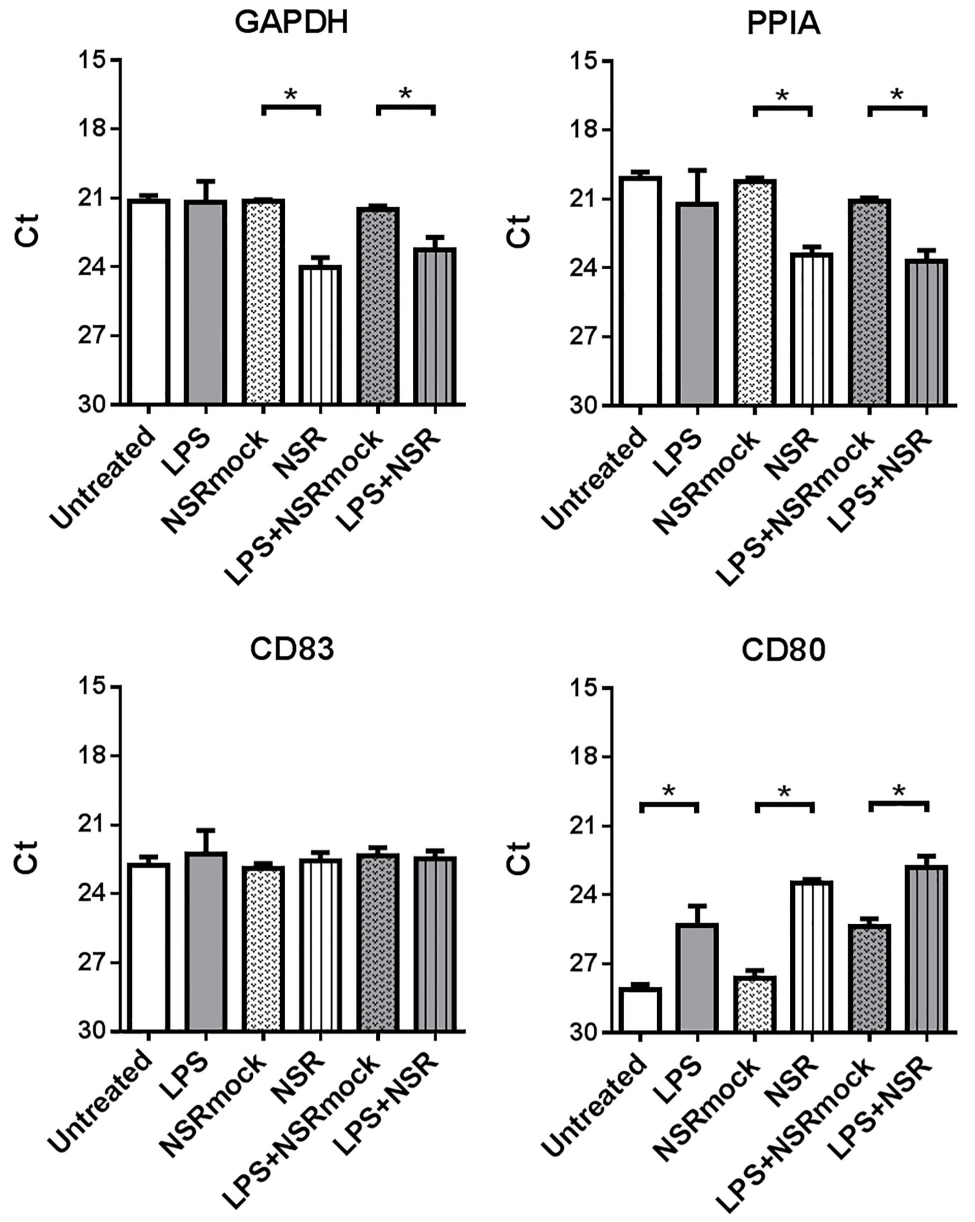
Analysis of CD83, CD80, GAPDH and PPIA mRNA levels. DC RNA samples, prepared 24 h after treatment (as indicated), were analysed by qRT-PCR. Bars represent average Ct values from triplicates ±SD with cells from one donor. The experiment is a representative of 3 independently performed experiments with cells from 3 different donors. Statistical significance is indicated with an asterisk.

To evaluate whether the reduced CD83 surface expression observed after NSR infection correlated with reduced mRNA levels, we analysed with qRT-PCR the quantities of CD83 mRNA in DC lysates prepared 24 h post NSR or LPS+NSR stimulation, resulting in more than 90% GFP-positive cells. Levels of two house-keeping gene mRNAs, glyceraldehyde-3-phosphate dehydrogenase (GAPDH) and peptidylprolyl isomerase A (PPIA), and the level of CD80 mRNA were determined as well and served as controls. The results show that neither NSR infection, nor a combination of LPS stimulation and NSR infection significantly affected the total levels of CD83 mRNA, as compared to control stimulations (Fig 5). In contrast, levels of CD80 mRNA were upregulated by both NSR and LPS+NSR stimulations, as well as by LPS stimulation alone, respective to the relevant controls. Remarkably, incubation of the DCs with NSR or LPS+NSR resulted in significant decreases in mRNA levels of GAPDH and PPIA.

**Fig 6:**
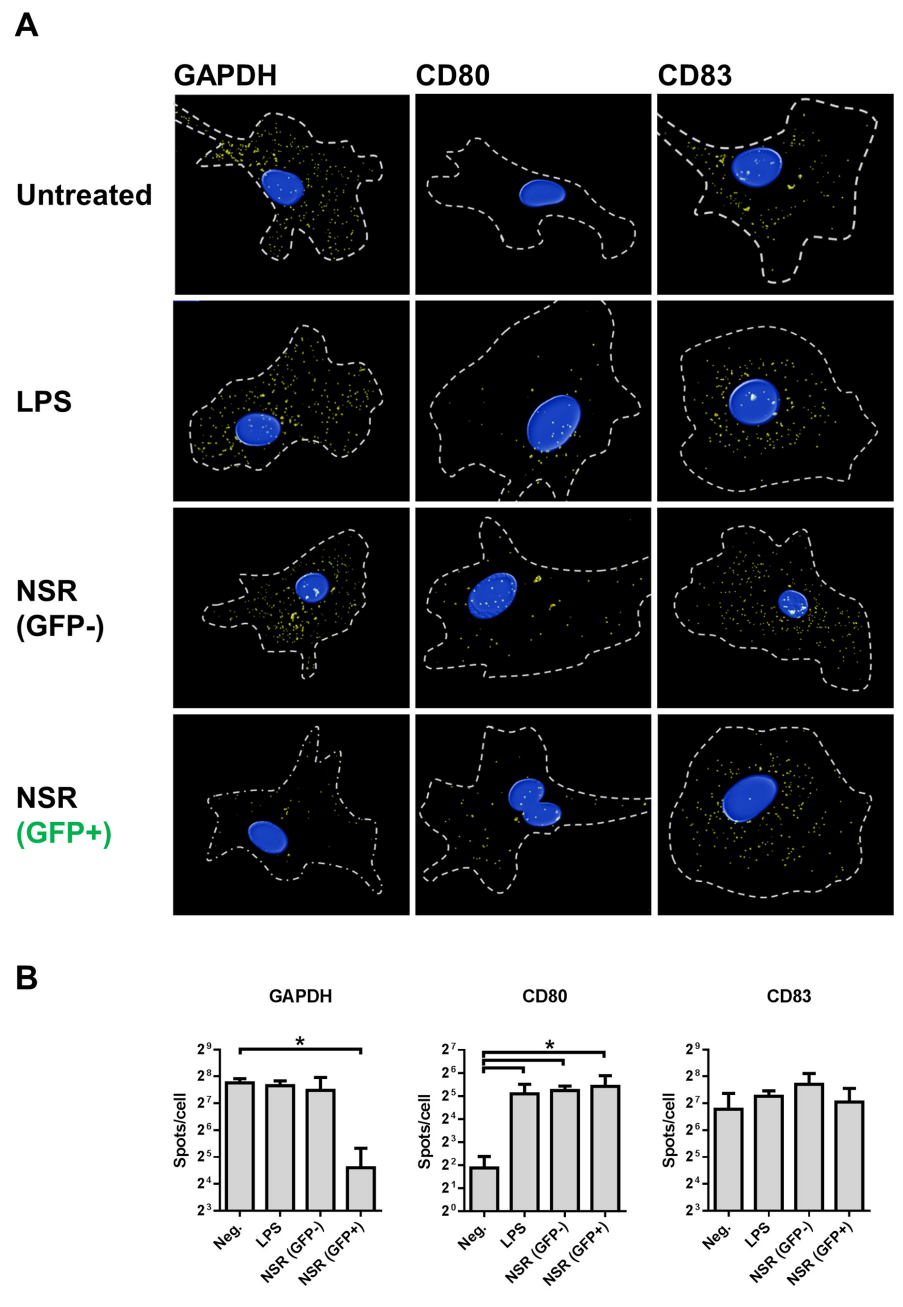
Visualization of GAPDH, CD80 and CD83 mRNAs in infected cells using FISH. DCs were stimulated for 24 h with LPS, infected with NSR or left untreated and then fixed and subjected to FISH. Shown are (A) representative cells of each treatment condition from three independently performed experiments with cells from three different donors and (B) average ±SD spot counts of cells probed for GAPDH, CD80 and CD83. Relevant statistical significance is indicated with an asterisk.

To be able to discriminate between GFP+ and GFP- in the DC population and to exclude possible arrest of host mRNA nuclear transport, which is a common mechanism used by viruses to counteract cellular antiviral mechanisms [29], we also evaluated subcellular location of GAPDH, CD80 and CD83 mRNAs using a fluorescence *in situ* hybridisation (FISH) technique. With this technique, individual mRNA molecules are visualized, revealing their total amount and cellular location. The overall FISH results corresponded very well with the qRT-PCR data (Fig 6). The decrease in the quantity of GAPDH mRNA, detected with qRT-PCR in NSR-infected cells, correlated with significantly reduced numbers of spots, detected with FISH in GFP+ cells. Interestingly, no reduction in GAPDH mRNA was observed in GFP- cells. CD80 mRNAs levels were induced by LPS stimulation, as well as by NSR infection, both in GFP+ and GFP- cells. Analysis of CD83 mRNA revealed similar total number of spots in all treatment conditions and no differences in mRNA distribution. To confirm the specificity of the FISH assay, we incubated DCs with LPS or a combination of LPS and Actinomycin D or Leptomycin B. The former drug suppresses cellular transcription, while the latter specifically blocks nuclear export via CRM1, an export route known to be utilized by CD83 mRNA [30–33]. As expected, treatment with Actinomycin D resulted in almost complete abrogation of CD83 mRNA production and treatment with Leptomycin B resulted in increased nuclear localization of this mRNA (see S1 Fig). Collectively, the FISH and qRT-PCR data reveal that the downregulation of the CD83 protein observed after NSR infection was not associated with downregulation of CD83 mRNA levels or arrest of CD83 mRNA transport from the nucleus.

### Downregulation of CD83 at the cell surface does not result from intracellular protein redistribution or increased release to the medium

**Fig 7:**
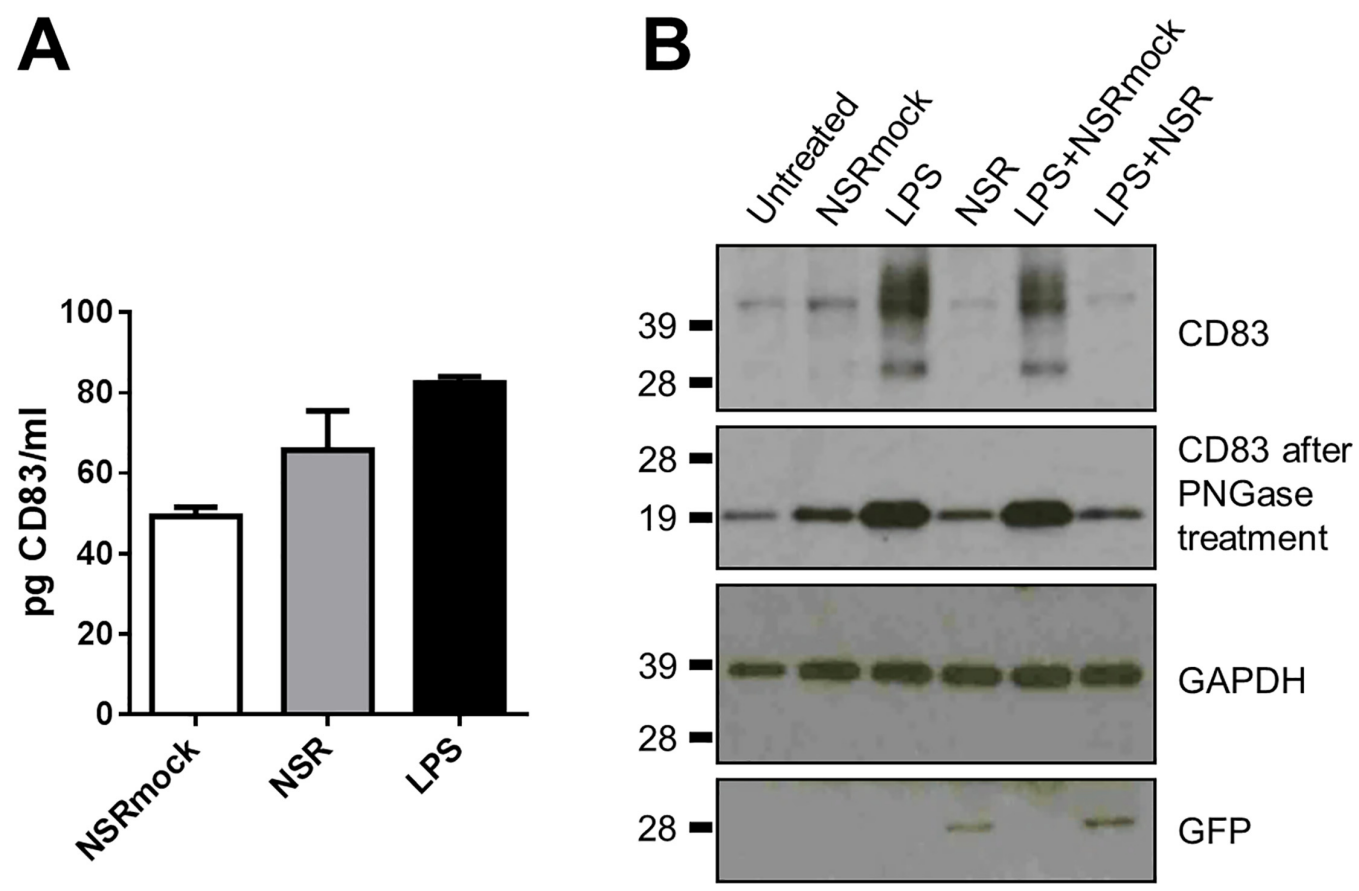
Effect of NSR infection on intracellular and extracellular CD83 levels. (A) The levels of soluble CD83 in supernatants from cells harvested 24 h after stimulation with LPS, infection with NSR, or from cells mock infected with NSRmock were determined by ELISA. Bars represent average CD83 concentrations ±SD. Results from one of two independently performed experiments with similar results are shown. (B) Detection of CD83 in cell lysates by Western blot at 24 hpi. The different treatments are shown above the top panel and the probed proteins are depicted at the right. The positions of molecular weight standard proteins are shown at the left. The top blot was stripped and re-probed with antibodies against GAPDH and GFP, which served as loading control and control to confirm NSR infection, respectively. Results from one of two independent experiments with cells from two donors are shown.

Sénéchal and co-workers reported downregulation of CD83 in monocyte-derived mature DCs infected with human cytomegalovirus [34]. The downregulation was attributed to release of the protein into the culture medium. This finding was confirmed by Kummer *et al* [35]. Regarding those reports, we investigated whether the downregulation of CD83 in NSR-infected cells resulted from increased shedding of the protein from the cell surface. To this end, supernatants of NSR-infected cells, NSRmock-infected cells and of cells stimulated with LPS were harvested at 24 hpi and analysed for the presence of soluble CD83 by ELISA. Quantities of CD83 in the supernatant of LPS stimulated cells were comparable to those previously reported (Fig 7A) [35]. Levels of soluble CD83 in culture media from NSR-infected cells did not statistically differ from those in culture media of NSRmock-infected cells or LPS-stimulated cells, demonstrating that shedding of CD83 into the growth medium does not explain the observed surface downregulation in NSR-infected DCs.

Since we did not detect increased levels of CD83 in culture media of NSR infected cells, we proceeded with analyses of intracellular CD83 protein levels. As CD83 expression was first induced and subsequently downregulated in NSR-infected cells, we hypothesized that these alterations may result from trapping of the molecule inside the cells. To test this hypothesis, we analysed with SDS-PAGE and Western blotting the quantities of CD83 in cell lysates of NSR-infected cells and lysates of LPS+NSR co-stimulated cells and compared these with CD83 quantities in unstimulated cells, cells stimulated with NSRmock, LPS or LPS+ NSRmock at 24 hpi. More than 90% of the DCs inoculated with NSR were positive for GFP. In unstimulated and NSRmock-stimulated cells, a single band of around 42 kDa was visible, corresponding to the known molecular weight of CD83 when present in intracellular protein pools [36] (Fig 7B). Upon induction with either LPS or LPS+NSRmock, CD83 expression was upregulated as evidenced by the appearance of a lower molecular weight band that corresponds to *de novo* synthesized CD83, as well as higher molecular weight bands that correspond to high-glycosylated, surface-exposed protein [36]. In both NSR and LPS+NSR stimulated cells, the detected CD83 protein levels closely resembled those in the unstimulated and NSRmock stimulated cells and only the band that corresponds to the preformed protein was visible, while the bands corresponding to the *de novo* form and the high-glycosylated form were not detected. Digestion of cell lysates with peptide-N-glycosidase F to remove the N-linked carbohydrates revealed a discrete band of deglycosylated protein. Amounts were comparable in unstimulated, NSRmock, NSR and LPS+NSR stimulated cells and much higher in cells stimulated with LPS or LPS+NSRmock. The total CD83 protein quantities in cell lysates correlated well with those measured by flow cytometry. This finding suggests that the observed downregulation of CD83 from the cell surface of NSR-infected cells at 24 hpi does not result from trapping of the protein inside the cells.

### Inhibition of the proteasomal or endocytic degradation pathways does not prevent NSR-mediated downregulation of CD83

**Fig 8:**
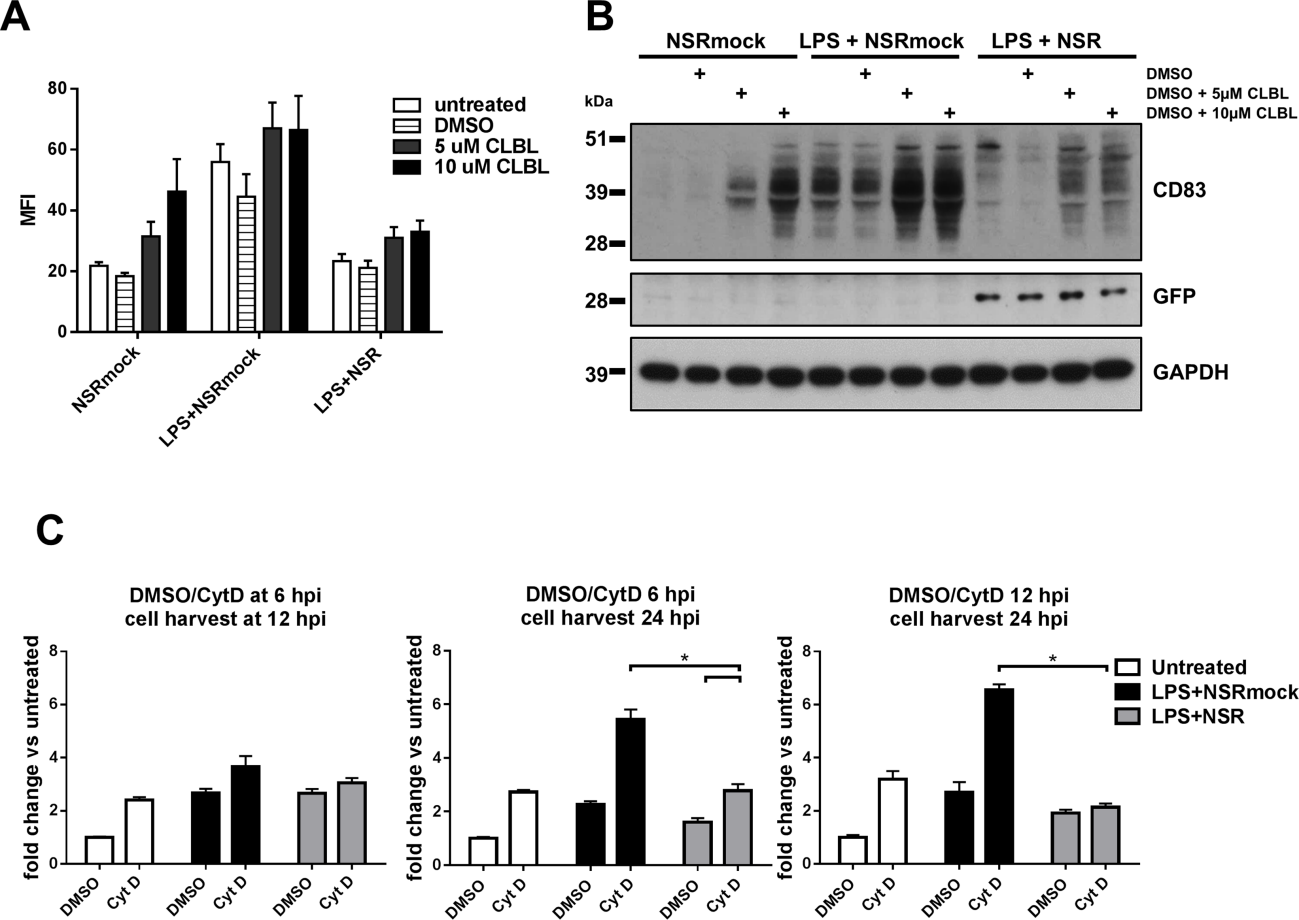
Expression of CD83 after inhibition of cellular protein degradation routes. (A) Flow cytometry analysis of CD83 surface expression after inhibition of the proteasome. DCs were stimulated with NSRmock, LPS+NSRmock or LPS+NSR for 8 h and then clasto Lactacystin β-lactone (CLBL) was added at two concentrations, as indicated. Control cells were left untreated or were treated with DMSO. Cells were analysed at 24 hpi for CD83 expression. Bars represent average MFI ±SD from two experiments with cells from one donor (B) Detection of total amounts of CD83 in cell lysates by Western blot. Cells were stimulated as described under point “A” and treatments are indicated above the image. (C) Inhibition of endocytosis. DCs were stimulated with LPS+NSRmock, LPS+NSR or left unstimulated. Cytochalasin D (Cyt D) or the solvent DMSO were subsequently added at different time points. The moments of adding Cyt D/DMSO and harvesting of cells are indicated above each graph. Bars represent average fold change of the MFI relative to unstimulated cells treated with DMSO ±SD. Average values of three experiments with cells from one donor are depicted. Relevant statistical significances are shown.

Mature human DCs, infected with herpes simplex virus type 1 (HSV-1), were reported to downregulate CD83 by proteasomal degradation [35]. This process is mediated by the immediate-early protein ICP0 of HSV-1 and is prevented by inhibition of the cellular proteasome machinery. To investigate whether proteasomal degradation of CD83 explains the decreased surface exposure of this molecule after NSR infection, we used the inhibitor clasto Lactacystin β-lactone (CLBL) to supress cellular proteasomal activity. This drug acts selectively and irreversibly on the 20S and 26S subunits of the proteasome without affecting serine and cysteine proteases. We preferred CLBL because earlier experiments demonstrated that this drug did not exert negative effect on RVFV replication, as opposed to another often used alternative drug, MG-132 [2]. As we already showed that CD83 levels increase in the first 8-12h after infection and then gradually decrease, we first stimulated DCs with NSRmock, LPS+NSR or LPS+NSRmock for 8 h and then added CLBL in two different concentrations. DMSO, the solvent of CLBL, was used as a control. Flow cytometry analysis was performed at 24 hpi. Addition of CLBL to NSRmock-infected cells resulted in a dose dependent increase of CD83 surface expression with a 70% increase for the lower CLBL concentration and with a 150% increase for the higher concentration, as compared to DMSO treatment (Fig 8A). In both LPS+NSRmock and LPS+NSR stimulated cells, a 50% increase in the surface expression of CD83 was measured that was not dependent on the CLBL dose, but the ratio of CD83 between the two treatment conditions remained unchanged. Total amounts of CD83 detected in cell lysates of LPS+NSR, LPS or NSRmock-stimulated cells after treatment with CLBL correlated well with the amounts of the surface expressed protein (Fig 8B). Together, these data suggest that NSR-infection does not result in increased proteasomal degradation of CD83.

In both immature and mature DCs, stable levels of CD83 at the DC surface are maintained by continuous production and recycling of the exposed protein [36]. Inhibition of endocytosis with cytochalasin D (Cyt D) was shown to promote surface exposure of CD83 in both DCs types. CytD is a drug that depolymerizes F-actin filaments and thereby blocks endocytosis, without affecting the exocytic pathway [37–39]. Cyt D treatment of infected cells was therefore employed to investigate possible involvement of endocytic degradation pathways in NSR-mediated downregulation of CD83. LPS+NSR co-stimulated cells, control LPS+NSRmock stimulated cells and unstimulated cells were incubated with Cyt D. Treatment with Cyt D for 6 h, added 6 h after stimulation resulted in an increase in surface-exposed CD83 in all stimulation conditions. However, in infected cells, this increase was the lowest (Fig 8C, left panel). Longer incubation with Cyt D (18 h) resulted in a further increase of CD83, but CD83 levels in cells co-stimulated with LPS+NSR were significantly lower than those in LPS+NSRmock stimulated cells (Fig 8C, middle panel). Importantly, when Cyt D was added at 12 h post treatment, it had no effect on CD83 expression levels in infected cells, while in control cells an increase in expression was observed (Fig 8C, right panel). Altogether, these data suggest that CD83 downregulation in NSR-infected cells does not involve increased degradation via the proteasomal or endocytic degradation pathways.

## Discussion

DCs express various pathogen recognition receptors that are able to sense viral RNA, including Toll-like receptors 3, 7 and 8, and cytoplasmic helicases RIG-I, MDA5 and LGP2 [40]. Of these receptors, RIG-I was shown to play a primary role in cytoplasmic detection of RVFV [41]. Upon recognition of its ligand, RIG-I triggers a signalling cascade resulting in activation of transcription factors NF-κB, IRF3 and 7 [42, 43], which induce cell maturation [44] and production of type-I interferons [45]. This whole cascade of events is purposed to control virus replication in the infected DC and to prepare the cell for efficient antigen presentation. A well-established mechanism to control virus dissemination from infected cells is apoptosis, which can be initiated by RIG-I activation [46]. Based on the aforementioned, we propose NSR-infected DCs succumb to apoptosis between 24 at 48 hpi resulting from RIG-I activation. In support of this hypothesis, infected cells displayed a significant decrease of GAPDH mRNA levels, which is known to be associated with early apoptotic events [47, 48].

Induction of apoptosis was previously reported to occur upon infection of DCs with replicon particles of the alphavirus Venezuelan equine encephalitis virus [49, 50], which have proven to be highly immunogenic [51]. Moreover, apoptosis of DCs was found to be essential for optimal efficacy of alphavirus replicon-based DNA vaccines in a tumor challenge mouse model. It is well documented that apoptotic cells can be taken up by DCs and serve as a source of antigen for cross-presentation [52], a mechanism that plays an important role in efficient priming of specific CD8+ responses [53, 54].

We observed that NSR-infected DCs did not fully mature, while bystander DCs did, as evidenced by CD83 upregulation. Maturation of bystander DCs is triggered by the presence of apoptotic infected DCs and cytokines and chemokines released as a result of the infection [55–57]. In supernatants of infected cells we detected IFN-β, TNF and IL-6, which are important for activation of antigen-presenting cells [58, 59] and are likely involved in the observed maturation of bystander cells. Additionally, type-I interferons were shown to augment the efficiency of cross-presentation [60]. Full maturation of bystander cells therefore suggests that these cells play a critical role in the priming of T-cell responses via cross-presentation of antigens, acquired from apoptotic infected cells. In accordance to this assumption, we hypothesize that GFP+ cells with restored levels of CD83 at 48 hpi represent mature bystander DCs that acquired GFP by phagocytosis of apoptotic NSR-infected DCs.

A surprising finding was the gradual downregulation of CD83 in NSR-infected DCs. Interestingly, NSR infection also prevented CD83 upregulation by LPS. Downregulation of CD83 is, however, not an unusual consequence of virus infection of DCs. In HIV-1 infected DCs, a reduced surface exposure of CD80, CD86 and CD83 was observed, which was shown to result from downregulation of the respective mRNAs, mediated by the Vpr protein [61]. Human cytomegalovirus infection of mature DCs was shown to cause a decrease in surface expression of CD83 which coincided with detection of a soluble form of this molecule in the cell culture medium [34]. Herpes simplex virus 1 infection of mature DCs resulted in rapid proteasomal degradation of CD83, mediated by the viral immediate-early protein ICP0 [35, 62]. In all cases, CD83 downregulation correlated with a reduced capacity to stimulate naïve T cells, revealing that targeting of CD83 is employed by these viruses as a specific means of immune evasion.

In NSR-infected cells, CD83 downregulation was not correlated with decreases in mRNA levels. The similar amounts of CD83 mRNA in DCs regardless of the maturation state are consistent with the mRNA pools previously described by Kruse *et al* [30]. Notably, the mRNA levels of two house-keeping genes were significantly downregulated in infected DCs, suggestive of a general suppression of cellular mRNAs, which can be attributed to early apoptotic events associated with degradation of cellular mRNA [47, 48]. Additionally, viral cap-snatching could be involved, resulting in destabilization of cellular mRNA and accelerated degradation. The unaffected CD83 mRNA levels and the induced levels of CD80 mRNA in infected DCs could be explained by increased stability of mRNAs that encode proteins dedicated to DC maturation and antigen presentation.

The lack of correlation between the stable mRNA levels and the reduction of CD83 in infected cells led us investigate whether CD83 downregulation occurs at the protein level. NSR infection did not result in increased amounts of CD83 in the cell culture medium. Analysis of cell lysates revealed that CD83 levels in NSR-infected cells were low and comparable to the levels in unstimulated cells. This finding suggested that in infected cells the protein was either not produced or very efficiently degraded. Involvement of enhanced degradation was investigated by using inhibitors of the major cellular protein degradation routes. Inhibition of the proteasomal protein degradation route did not restore levels of CD83 in NSR-infected DCs. Inhibition of endocytosis at early time points after infection (6 hpi) only partially restored CD83 levels, while inhibition at later time points (12 hpi) did not result in restoration of CD83 levels. These data demonstrate that downregulation of CD83 does not result from proteasomal degradation or lysosomal degradation following endocytosis. Considering all our findings, we propose that CD83 in NSR-infected cells is downregulated at the translational level. This notion is further supported by the kinetics of CD83 downregulation. CD83 downregulation occurs relatively slowly, after an initial upregulation that peaks between 8 and 12 hpi. In contrast, HSV-1-mediated downregulation that was shown to depend on proteasomal degradation was already significant within 10 hpi. The initial upregulation after NSR infection can be explained by the presence of protein and mRNA pools of CD83 in immature DCs that are ready to be mobilized upon stimulation [30, 36, 63]. Thus, we propose a model where infection with NSR is sensed by DCs, resulting in surface exposure of CD83 originating from intracellular pools. As viral replication and protein synthesis progresses, an unknown viral and/or cellular factor inhibits translation of CD83. CD83 originating from the protein pools is recycled normally but not replenished, resulting in relatively slow downregulation.

In summary, we demonstrate that NSR infection of monocyte-derived immature DCs results in incomplete maturation, associated with gradual downregulation of CD83. The observed downregulation is attributed to inhibition at the translational level. Bystander cells reached a fully matured phenotype, which suggests that these cells play a central role in NSR-mediated immunity. Considering our findings, it is interesting to speculate about the importance of DC targeting and cross presentation in NSR-mediated immunity, which we plan to address in future studies.

## Supporting Information

S1 Fig Evaluation of the specificity of CD83 FISH assay. DCs were stimulated with LPS or co-incubated with LPS and Actinomycin D or LPS and Leptomycin B for 24 h and then probed for CD83 mRNA. Shown are representative cells from the respective treatments. Cells from one donor were used.
(TIF)

S1 Table Sequences of the primers used for quantification of CD83, CD80, GAPDH and PPIA mRNAs by real-time PCR. (DOCX)

S2 Table Sequences of the probes used for fluorescence in situ hybridization (FISH). (DOCX)
