# Supplementary figures and images for: Nonspreading Rift Valley Fever Virus Infection of Human Dendritic Cells Results in Downregulation of CD83 and Full Maturation of Bystander Cells
Source: PLoS One. 2015 Nov 17;10(11):e0142670. doi: 10.1371/journal.pone.0142670 (PMC4648518; doi:10.1371/journal.pone.0142670)

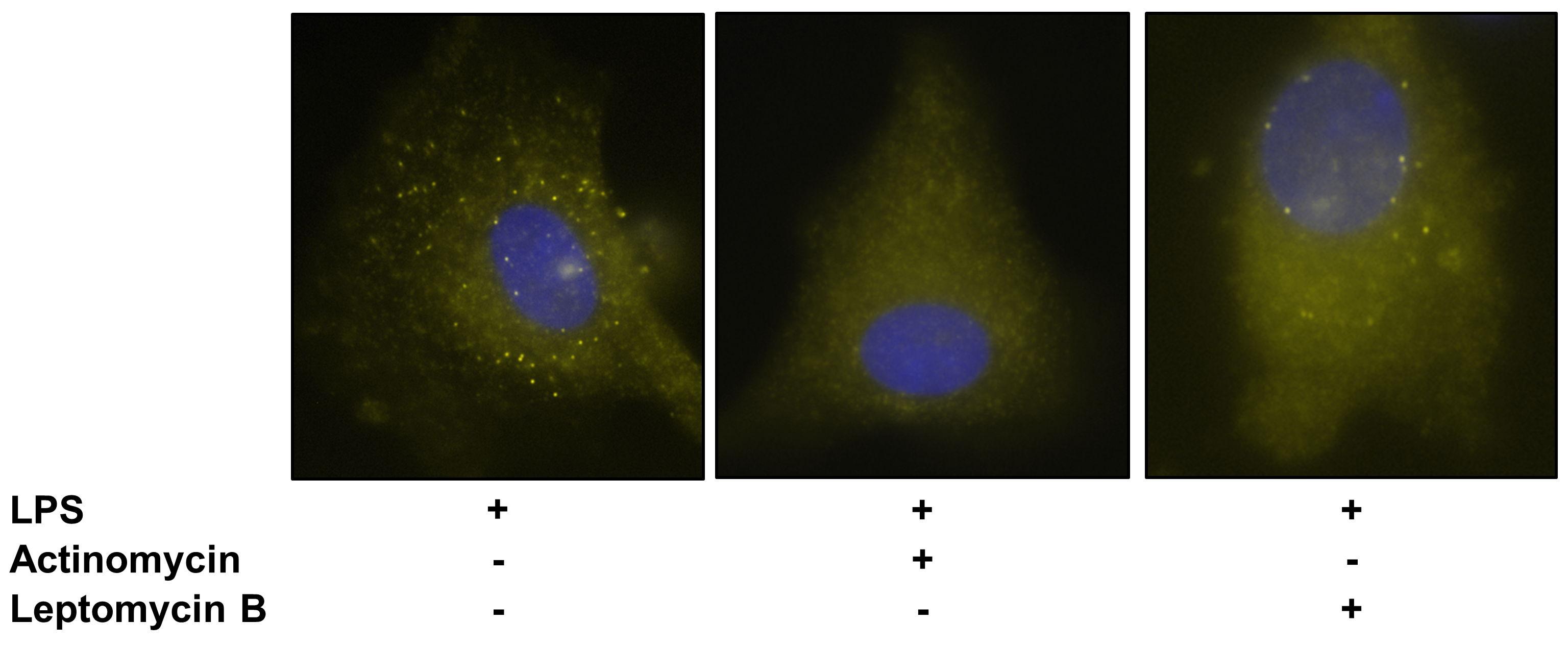

Supplement: S1 Fig — DCs were stimulated with LPS or co-incubated with LPS and Actinomycin D or LPS and Leptomycin B for 24 h and then probed for CD83 mRNA. Shown are representative cells from the respective treatments. Cells from one donor were used. (TIF) [file pone.0142670.s001.tif]
