# Supplementary material for: Nonspreading Rift Valley Fever Virus Infection of Human Dendritic Cells Results in Downregulation of CD83 and Full Maturation of Bystander Cells
Source: PLoS One. 2015 Nov 17;10(11):e0142670. doi: 10.1371/journal.pone.0142670 (PMC4648518; doi:10.1371/journal.pone.0142670)
Supplement: S1 Table — (DOCX) [file pone.0142670.s002.docx]

S1 Table

Sequences of the primers used for quantification of CD83, CD80, GAPDH and PPIA mRNAs by real-time PCR

| **mRNA** | **Forward primer** | **Reverse primer** |
| --- | --- | --- |
| CD83 | ATGGAGACACCCCAGGAAGAC | TCAGGGAATAGGGCCTTTCA |
| CD80 | CCTCAATTTCTTTCAGCTCTTGGT | GGACAGCGTTGCCACTTCTT |
| PPIA | CATACGGGTCCTGGCATCTT | TGCCATCCAACCACTCAGTCT |
| GAPDH | CCATCTTCCAGGAGCGAGATC | GCCTTCTCCATGGTGGTGAA |
