# Supplementary material for: Nonspreading Rift Valley Fever Virus Infection of Human Dendritic Cells Results in Downregulation of CD83 and Full Maturation of Bystander Cells
Source: PLoS One. 2015 Nov 17;10(11):e0142670. doi: 10.1371/journal.pone.0142670 (PMC4648518; doi:10.1371/journal.pone.0142670)
Supplement: S2 Table — (DOCX) [file pone.0142670.s003.docx]

S2 Table

Sequences of the probes used for fluorescence *in situ* hybridization (FISH)

| **mRNA** | **Probe** |
| --- | --- |
| GAPDH | Human GAPDH (Stellaris FISH probes, SMF-2026-1) |
| CD83 | ctcaggagcagaagctggag caggagaccgtgtagggaac accaccctccaataacttga tttgccccttctgatgatag attgggggcgtcgaaagaac ctgcagagtgcacctgtatg aagtctcttctttacgctgt acaatctccgctctgtattt ctgggaagatactctgtagc tccatgccagctttagaaaa cactagccctaaatgcttat ataccagttctgtcttgtga agaacctgcagaaatcctgc cagtgtaacagacaggcaca ttcagcgtaggctcattctt tgaaggacttcacaggatgc cttccagatgttttcagtga ccacagaaaatggggtggga atgtggtcatgtgatggttt agctgcatacatcgctgaaa catctcaccatagcttttat atatttcccaagaccctttt gatgcagaagaggacagctg aaacatctgggctggtacag cttgatctgtcaatttctcc tccttagtaatagcaggacc ggggaatagttcatcgactt tttcccagatgaaaagaccc ctcttctttattgcttcttt gtcagttcacaatgctatct acttcttctccctctctatg ggccattaaatagctctcta atgactcaatggagtttcga caccatcatcatagcaagga tgagaaatggatagcaccct ttcttggtaaccttctttgt cctacagaaggctacaggaa cttcaagaacagaccgtgga ccatatcttggagtgtgtta aacatccatgcaacacttcg agggaaagccaacaatggct caagaagggaagggcccaag cctaatattagggggacagt atcaacttggtatccgtttt atgaaagccaagacagaggt ctttcttcacatgcatacgt |
| CD80 | gagttcactcagtacttgtc tctgttactttacagagggt atcttcagagaggcgacatt gtggatttagtttcacagct gggctgatgacaatccaatt aaagccaacaatttggaccc cttcagatgcttagggtcaa gaggtatggacacttggatg gaagtgagaaagaccagcca tgccagctcttcaacagaaa caaagatggtccggttcttg cgcttgaaagcgtctttttc tgtagggaagtcagctttga tggaaaacctccagaggttg ctgttgtgttgatggcattt agcatagagctcagtttcag tgaaatccagtttgctgcta gagacacatgaagctgtggt tccagttgaaggtctgattc ggaaaatgctcttgcttggt gtcaggcagcatatcacaaa ctgcggacactgttatacag ggaccttcagatcttttcag aagttcccagaagaggtcaa caaggtggggtaatcttgtc agcacctaagagcagatacg agacacttcctgcaaagcaa cttgatcaaggtcaccagag gtattctctaaagtcccttc gggaaagagcaccagagtta aggccacagtcaaactgata aaattctacttccagcagca tctctgaagttgacctgtta ctatgcattacattgggctt tcctaaagatgttcatgcca tcgtcttacatgtcagagga cttgactactgctttgacgt ttaagtctttggactatccc ccctatggaaagttactacc ccatcttagggatctaagga acccagaaataccaaggaga gctggctctaaaggctttaa tagtgaggtagctaaagcca aggagcaaggtttgtgaagc ctgccctacactgagaatat ggaaacactgctagtacctt gctgacaaagtatctgctgt caactttgtttcttccctta |
